# Stigma, medication adherence and coping mechanism among people living with HIV attending General Hospital, Lagos Island, Nigeria

**DOI:** 10.4102/phcfm.v4i1.417

**Published:** 2012-11-13

**Authors:** Adekemi O. Sekoni, Obinna R. Obidike, Mobolanle R. Balogun

**Affiliations:** 1 Department of Community Health and Primary Care, University of Lagos, Nigeria; 2 Massey Children Hospital, Lagos Island, Nigeria

## Abstract

**Background:**

People living with HIV and AIDS (PLWHA) experience some form of stigma which could lead to poor medication adherence.

**Objectives:**

This study assessed the various domains of stigma experienced by PLWHAs attending an HIV clinic at General Hospital, Lagos Island, their medication adherence patterns and their coping mechanisms for ensuring adherence to antiretroviral therapy.

**Method:**

A cross-sectional study design with a sample size of 200 was used. Respondents were selected using systematic random sampling. Interviewers administered structured questionnaires were used to collect information on the domains of stigma. Data was analysed using EPI info^©^. This was followed by a focus group discussion (FGD) with seven participants at the clinic using an interview guide with open-ended questions.

**Results:**

Overall, stigma was experienced by 35% of the respondents. Within this group, 6.6%, 37.1%, 43.1% and 98.0% of the respondents reported experiencing negative self image stigma, personalised stigma, disclosure stigma and public attitude stigma respectively. Almost 90% of the respondents were adherent. The FGD revealed that disclosure was usually confined to family members and the coping mechanism for achieving adherence was to put antiretroviral (ARVs) in unlabelled pill boxes.

**Conclusion:**

This study found that stigma was low and that the most common domain of stigma experienced was public attitude stigma. Medication adherence of respondents was good as a result of the coping mechanism, which involves putting ARVs in unlabelled pill boxes.

## Abstrait

**Stigmatisation, observance du traitement et mécanismes de gestion chez les personnes vivant avec le VIH traitées à l'hôpital général de l’île de Lagos au Nigéria**

**Contexte:**

Les personnes vivant avec le VIH et SIDA (PVVS) connaissent des formes de stigmatisation qui peuvent entraîner une mauvaise observance de leur traitement.

**Objectifs:**

Cette étude évalue les différents types de stigmatisation vécus par les personnes vivant avec le VIH/SIDA traitées au centre de traitement du VIH de l'hôpital général de l’île de Lagos, leurs niveaux d'observance du traitement et leurs mécanismes d'adaptation pour assurer l**’**observance de la thérapie antirétrovirale.

**Méthode:**

Une étude transversale a été réalisée sur un échantillon de 200 personnes. Les participants ont été sélectionnés de manière aléatoire et systématique. Les enquêteurs ont utilisé des questionnaires structurés afin de recueillir des informations sur la stigmatisation. Les données ont été analysée par EPI info^©^. Des groupes de discussion thématique ont été ensuite mis en place avec sept participants au centre de traitement, en utilisant un guide d'entretien avec des questions ouvertes.

**Résultats:**

Au total, 35% des personnes interrogées se sont dites victimes de stigmatisations. Au sein de ce groupe, 6.6%, 37.1%, 43.1% et 98.0% des personnes interrogées ont indiqué ressentir respectivement une stigmatisation associée à une mauvaise image de leur propre personne, une stigmatisation de la personne, une stigmatisation associée à la divulgation de leur statut et associée à l'attitude du public. Près de 90% des personnes interrogées observaient leur traitement. Le groupe de discussion a révélé que la divulgation de la maladie se limitait essentiellement à la famille et que le meilleur mécanisme d'adaptation pour observer leur traitement était de placer les cachets de traitement antirétroviral (ARV) dans des piluliers non étiquetés.

**Conclusion:**

Cette étude a révélé que la stigmatisation était faible et que la plus importante était celle ressenti par rapport à l'opinion publique. Les personnes interrogées suivent leur traitement grâce au mécanisme d'adaptation qui consiste à placer les comprimés d'ARV dans des piluliers non étiquetés.

## Introduction

### Setting

Stigma and unfair discrimination associated with HIV infection occurs globally as a result of poor knowledge of its transmission, fears relating to illness and incurable diseases, inadequate access to treatment and religious beliefs. According to the United Nations declaration of commitment on HIV and AIDS, stigma, silence, discrimination and denial, as well as lack of confidentiality, undermine efforts aimed at prevention, care and treatment thereby increasing the impact of the epidemic on individuals, families, communities and nations.^1^

#### Key focus

Stigma has been described as an undesirable or discrediting attribute that an individual possesses which reduces the status of that individual in the eyes of society whilst discrimination is a negative act that results from stigma.^2^ In view of the fact that it could become an obstacle preventing PLWHAs from accessing healthcare services and achieving medication adherence, the theme of the World AIDS Day campaign in 2002 and 2003 focused on reducing HIV-related stigma and discrimination.^1^ According to the World Health Organization 2008 report, fear of stigma and discrimination was cited as one of the reasons why uptake of client initiated HIV counseling and testing approach has remained limited.^3^

#### Background

From the time HIV was discovered, social responses of fear, denial, stigma and discrimination have accompanied it.^4^ The stigmatisation of individuals infected and affected by HIV and the eventual discrimination which they suffer are the tragic consequences of HIV disease. Stigma in the context of HIV tends to create a hidden epidemic of the disease based on socially-shared ignorance, fear, misinformation and denial.^5^ The issues of stigma and discrimination described by Jonathan Mann as the third phase of the HIV pandemic (the first two being the hidden but accelerating spread of HIV and the visible rise of AIDS cases) poses a serious threat to prevention and treatment.^6^ Because stigma hampers society's ability to respond effectively to HIV infection, understanding and counteracting it will remain a critical public health issue in any country. For current HIV prevention initiatives and treatment adherence in Nigeria to be effective, there is need for research to illuminate the cultural context of AIDS stigma in Nigeria.^7^

#### Trends

In 2008, a national survey conducted in Nigeria showed a substantial increase in the number of people (men and women) with an accepting attitude towards PLWHA compared to a similar study done in 2003. More respondents were willing to take care of a family member with HIV and AIDS in their home and keep secret the HIV positive status of a family member.^8^ In a study conducted in Cape Town, 40% of persons with HIV and AIDS had experienced discrimination resulting from having HIV infection and one in five had lost a place to stay or a job because of their HIV status. More than one in three participants indicated feeling dirty, ashamed, or guilty because of their HIV status.^9^ PLWHA in five African countries reported extensive verbal and physical abuse and neglect or negating (disallowing of access to services and opportunities), which was observed or documented by nurses caring for them. All of this represents the negative consequences experienced by PLWA whose HIV-positive status was disclosed to family, friends, or community members.^10^

Medication adherence plays a very important role in the survival and quality of life of PLWHA, thus it is imperative to study the effect of stigma on medication adherence amongst PLWHA in the Nigerian context. The impact of stigma on medication adherence by PLWHA who are on HAART cannot be overemphasised. In a study with 25 adolescents and young adults, half of the participants had missed medication doses for fear of disclosing their status and possible subsequent stigma.^11^

#### Objective

This study assessed the various domains of stigma amongst PLWHAs attending HIV Clinic at a PEPFAR supported treatment site in Lagos Nigeria, determined the effect of stigma on medication adherence and the coping mechanism used by the PLWHA for achieving medication adherence.

#### Contribution to field

The need to measure and assess stigma and its effect in the Nigerian context was the rationale behind this study. Most studies are only concerned with assessing a particular domain of stigma instead of assessing all the possible domains of stigma. This study tried to close the gap by looking at the four domains, (1) personalised stigma, (2) negative self image stigma, (3) disclosure stigma and (4) public attitude stigma, thereby providing a more comprehensive understanding of stigma associated with HIV which will assist programme managers in developing strategies for curbing or controlling it and consequently develop ways of mediating it in Nigeria and other countries in sub-Saharan Africa.

This work provides deeper insight into HIV infection-associated stigma experienced by people living with the virus, thereby helping to explore ways of addressing the problem and identifying a coping mechanism practiced that appears to promote ARV medication adherence.

## Ethical considerations

The proposal for the study was sent to the Lagos State Hospital Management Board and written permission was obtained before commencement of the study.

### Potential benefits and hazards

A questionnaire was administered to willing participants following informed verbal consent. There was no potential risk to the participants as names and addresses were not collected or documented and privacy was ensured during information collection.

### Recruitment procedures

Participants were patients who were registered and attend the HIV clinic at General Hospital, Lagos Island, Lagos. Participation was voluntary and no inducement in any form was given.

### Informed consent

Verbal informed consent was sought from the participants before questionnaire was administered and the focus group discussion (FGD) conducted.

### Data protection

Data generated from the study was coded so that the identities of the participants were not revealed and the data was stored in a secure location.

## Methods

### Materials

A structured, pre-tested, interviewer-administered questionnaire was used for collecting data. The contents were grouped as sections (A) Socio-demographic Information, (B) Medication Adherence Assessment, (C) Stigma Assessment. Sections A and B were developed by the researcher based on information gathered during the review of literature whilst section C was adapted from the Stigma Scale developed by Berger and colleagues.^12^

### Setting

General Hospital, Lagos Island, is a secondary healthcare institution that provides health care services to all segments of society. It operates some of the major disciplines of the medical profession, which include departments of internal medicine, surgery, dental care, an eye clinic, an ear, nose and throat clinic, a pharmacy and a laboratory. The HIV clinic was managed by Médecins Sans Frontières (MSF) in 2004; they provided free comprehensive care for PLWHA until 2008 when it was handed over to the hospital management who then partnered with the Global HIV and AIDS Initiative Nigeria (GHAIN) to continue providing care to PLWHAs. The clinic is structured such that patients are provided all the necessary services without the need to go to the main hospital building. Mondays, Tuesdays and Fridays are clinic days and services are provided from 8h00 to 16h00. There were 3017 registered patients with the HIV clinic as at February 2011. The clinic witnesses an average turnout of 200 patients per clinic day. An admissions facility was also available.

### Design

A descriptive cross-sectional study (with quantitative and qualitative components) was carried out to assess the domains of stigma experienced by people living with HIV and AIDS attending the HIV clinic at General Hospital, Lagos Island. The calculated sample size was 200.

### Procedure

The study was carried out between April and May, 2011. Systematic random sampling was used to select the patients who were included in the quantitative survey each clinic day. There were 3017 patients registered with the clinic and this was used as the sampling frame. The sampling interval was calculated by dividing the sampling frame by sample size ([i.e. K = *N*/*n*]: 3017/200 = 15.085). On each clinic day, balloting was done to select the first sample unit amongst the first fifteen patients to arrive at the clinic and subsequent patients were selected at intervals of fifteen. If the selected patient happened to have been selected in previous weeks or refused to partake in the study, he or she was skipped and the next patient was selected.

An interview guide in English with open-ended questions was used to conduct the FGD, which took place in a conducive environment at the clinic on a non-clinic day after questionnaire administration had been completed. The participants for the FGD were purposively selected amongst the PLWHAs accessing services at the treatment site. Verbal informed consent was sought from the seven participants prior to the event, and the purpose of the FGD and the type of information needed was also explained. In the welcome address, participants were encouraged to communicate and interact with each other and told that the session would be tape-recorded. Any question not understood by the participants was interpreted by the research assistants in the local dialect. A ‘note taker’ also took notes. At the end of the one-hour session, the principal investigator thanked the participants and they were given refreshments. Information collected was analysed manually.

### Analysis

Data entry and analysis was done using Epi Info 2002. Frequency tables were generated for the category variables. Summary statistics were used for numerical variables. A scoring system was used to quantify the various domains of stigma.

Respondents were asked whether they agreed or disagreed with 16 items, and answer choices were offered on a four-point Likert scale (Strongly Disagree [0], Disagree [1], Agree [2], Strongly Agree [3]).^13^ The 16 items, which was developed by Berger and colleagues,^12^ assessed the four domains of stigma (each domain has 4 items), with a maximum score of 12 and a minimum of 0 for each domain of stigma. Percentage scores greater or equal to 50% (6 and above out of 12 scores) were considered to indicate that stigma was experienced, whilst those who had a percentage score of less than 50% (5 and below out of 12) were perceived not to have experienced stigma.^14^

Overall stigma was assessed based on the total of 16 items, which gave a maximum score of 54 and a minimum score of 0. Percentage scores greater than or equal to 50% (27 and above, out of 54) were considered to indicate that stigma was experienced, whilst those who had a percentage score of less than 50% were perceived not to have experienced stigma (scores of 26 and below, out of 54).^13^

Respondents were also asked some questions assessing their medication adherence. Seven days recall of medication usage by patients was used to measure medication adherence. Patients were asked if they had adhered to their medication within the last 7 days, and the total number of days they adhered was divided by 7 days and converted to a percentage.^15, 16, 17^ A score of 95% and above represented good adherence and less than 95% was rated as poor adherence.^18^ Socio-demographic characteristics and medication adherence attributes of PLWHA were assessed based on the various domains of stigma they experienced. A Chi-square statistical test was used to test the level of significance at a *p*-value of 0.05 whilst the FGD was analysed manually.

## Results

Out of the 200 interviewer-administered questionnaires, 197 were correctly filled in and analysed (98.5% response rate).

Most respondents were between 25 and 44 years of age (69.0%), and the mean age was 35.75 years ± 10.74. Female respondents were 64.5%, and 86.3% of respondents had at least secondary school education, of which 45.2% had tertiary education. Most of the respondents (42.6%) were married and there were more Christians (56.9%) and members of the Yoruba ethnic group (Table 1).

**TABLE 1:** Socio-demographic characteristics.

Characteristics	*n* = 197	%
**Age (yrs)**		
15-24	22	11.2
25-34	86	43.6
35-44	50	25.4
45-54	25	12.7
55-64	14	7.1
**Sex**		
Female	127	64.5
Male	70	35.5
**Educational level**		
No formal education	4	2
Primary	23	11.7
Secondary	81	41.1
Tertiary	89	45.2
**Marital status**		
Single	78	39.6
Married	84	42.6
Divorced and/or Separated	16	8.1
Widowed	19	9.7
**Religion**		
Christianity	112	56.9
Islam	84	42.6
Traditional	1	0.5
**Ethnic group**		
Yoruba	81	41.1
Igbo	46	23.4
Hausa	33	16.8
Efik	22	11.1
Ijaw	15	7.6

*n*, Given as number.

Respondents who had been attending the HIV clinic for over two years were 43.1%, whilst 40.1% had been attending for between six months and two years and 16.8% had been attending the clinic for less than six months. Respondents who had known their HIV status for over two years were 58.4%, whilst 34.5% had known for between 6 month to 2 years and 7.1% had known for less than six month. Respondents on ARV drugs were 71.6% whilst those not yet on ARV drugs were 28.4%. Out of the 141 respondents on ARV drugs, 25.5% had missed their doses whilst 74.5% had not (Table 2).

**TABLE 2:** Respondents on antiretroviral and those who missed doses.

Variables	*f*	%
**Respondents on ARV**		
Use ARV Drugs	141	71.6
Not Using ARV Drugs	56	28.4
**Total**	**197**	**100**
**Respondents who missed doses**		
Yes	36	25.5
No	105	74.5
**Total**	**141**	**100**

*f*, Frequency; ARV, antiretroviral.

Amongst respondents who missed their ARV medication, slightly more than half skipped doses because they got tired of their drugs (52.2%), 17.3% due to the presence of someone when it was time to take their medication, 13.1% because they had travelled without their medication whilst 13.1% because they felt better and the remaining 4.3% because of side effects.

There was no statistically significant association between the reasons why respondents missed medication and the frequency of missed ARV medication. Of the respondents who missed their medication when they travelled, most (66.6%) did so at least once in two months. Half of the respondents who skipped their drugs when they felt better, or because of someone's presence, did so at least once in two months. All respondents who had skipped doses in the past due to side effects did so at least once in two months. Also, 37.5% of those who got tired of taking the medication did so at least once in two months. Based on seven days recall, most respondents (89.4%) were adherent to their ARV medication, whilst 10.6% were not (Table 3).

**TABLE 3:** Medication adherence amongst respondents on antiretroviral.

Adherence	*f*	%
Non Adherent	15	10.6
Adherent	126	89.4

**Total**	**141**	**100**

*f*, Frequency.

Almost all of the respondents (98%) had experienced public attitude stigma, 43.1% of the respondents experienced disclosure stigma, 37.1% of the respondents experienced personalised stigma whilst a few of the respondents (6.6%) had experienced negative self image. Respondents who experienced overall stigma were 35% whilst 65% were perceived not to have experienced overall stigma.

There was a statistically significant association between respondents’ educational level, ethnic group and personalised stigma (*p* = 0.018 and 0.043 respectively). Respondents with the highest level of education (tertiary education) experienced the least personalised stigma (29.2%) and as the education level decreased from secondary to primary and finally to no formal education, personalised stigma increased from 40.7% to 43.5% and finally to 100%. Hausa respondents experienced the highest personalised stigma (54.5%), this was followed by Igbos (41.3%) and then 35.8% amongst Yorubas (Table 4).

**TABLE 4:** Relationship between socio-demographic characteristics and personalised stigma.

Socio-demographic characteristics	Personalised stigma				Total (*n* = 197)	Statistics
	No perceived stigma		Experienced stigma			
				
	*f*	%	*f*	%		
**Level of education**						
No formal Education	0	0.0	4	100	4	χ^2^ = 10.020
Primary	13	56.5	10	43.5	23	*df* = 3
Secondary	48	58.3	33	40.7	81	*p* = 0.018†
Tertiary	63	70.8	26	29.2	89	-
**Ethnic group**						
Yoruba	52	64.2	29	35.8	81	χ^2^ = 9.969
Igbo	27	58.7	19	41.3	46	*df* = 4
Hausa	15	45.5	18	54.5	33	*p* = 0.043†
Efik	18	81.8	4	18.2	22	-
Ijaw	12	80.0	3	20.0	15	-
**Medication use**						
Use ARVs	80	56.7	61	43.3	141	χ^2^ = 8.192
Do not Use	44	78.2	12	21.8	56	*p* = 0.004
**Duration of attendance**						
<6months	29	87.9	4	12.1	33	χ^2^ = 16.052
6months–2yrs	53	67.1	26	32.9	79	*df* = 2
>2yrs	42	49.4	43	50.6	85	*p* = 0.000†

χ^2^, Chi-square; *n*, Given as number; *f*, Frequency; ARVs, antiretroviral; *df*, Degree of freedom.

† fishers exact.

There was also a statistically significant association between ARV usage and personalised stigma (*p* = 0.004). More respondents (43.3%) on ARV experienced personalised stigma compared to 21.8% of respondents who were not on ARV. There was also an observed relationship between duration of clinic attendance and personalised stigma, with 50.6% of respondents who had been attending the clinic for over 2 years experiencing personalised stigma compared to 12.1% of respondents who had been attending the clinic for less than 6 months (Table 5).

**TABLE 5:** Relationship between medication use, duration of clinic attendance and personalised stigma.

Treatment history	Personalised stigma				Total (*n* = 197)	Statistics
			
	No perceived stigma		Experienced stigma			
				
	*f*	%	*f*	%		
**Medication use**						
Use ARVs	80	56.7	61	43.3	141	χ^2^ = 8.192
Do not Use	44	78.2	12	21.8	56	*p* = 0.004
**Duration of attendance**						
<6months	29	87.9	4	12.1	33	χ^2^ = 16.052
6months–2yrs	53	67.1	26	32.9	79	*df* = 2
>2yrs	42	49.4	43	50.6	85	*p* = 0.000†

χ^2^, Chi-square; *n*, Given as number; *f*, Frequency; ARVs, antiretroviral; *df*, Degree of freedom.

† fishers exact.

There was a statistically significant association between educational level and disclosure stigma (*p* = 0.019). Respondents who had primary education as their highest level of education experienced more disclosure stigma (56.5%) compared to PLWHAs with tertiary education (31.5%). Married respondents experienced more disclosure stigma (54.8%), as well respondents who had been aware of their status for between six months to two years (28.6%) (Table 6).

**TABLE 6:** Relationship between socio-demographic characteristics and disclosure stigma.

Socio-demographic characteristics	Disclosure stigma				Total (*n* = 197)	Statistics
			
	No perceived stigma		Experienced stigma			
				
	*f*	%	*f*	%		
**Education**						
No formal Education	2	50.0	2	50.0	4	χ^2^ = 9.211
Primary	10	43.5	13	56.5	23	*df* = 3
Secondary	39	48.1	42	51.9	81	*p* = 0.019†
Tertiary	61	68.5	28	31.5	89	-
**Marital status**						
Single	50	64.1	28	35.9	81	χ^2^ = 8.747
Married	38	45.2	46	54.8	84	*df* = 3
Divorced and/or Separated	12	75.0	4	25.0	16	*p* = 0.033†
Widowed	12	63.2	7	36.8	19	-

χ^2^, Chi-square; *n*, Given as number; *f*, Frequency; *df*, degree of freedom.

† fishers exact.

There was no statistically significant association between stigma and medication adherence but PLWHAs who did not experience overall stigma were more adherent to their medication (90.8%) compared to those who experienced overall stigma (87.0%) (Table 7).

**TABLE 7:** Relationship between overall stigma and medication adherence.

Stigma	Medication adherence				Total	Statistics
			
	Non adherent		Adherent			
				
	*f*	%	*f*	%		
No perceived stigma	8	9.2	79	90.8	87	χ^2^ = 0.497
Experienced stigma	7	13.0	47	87.0	54	*p* = 0.481

**Total**	**15**	**10.6**	**126**	**89.4**	**141**	**-**

χ^2^, Chi-square; *f*, Frequency.

### The Focus Group Discussion

The PLWHAs were of the opinion that poor knowledge about HIV and AIDS sometimes results in people asking them embarrassing questions when they disclose their status and the denial of medical services from healthcare workers.

‘Lack of understanding by the people within the society. The PLWHAs here understand everything about the virus but the people in the community do not have the understanding and this is what leads to stigma.’ (Participant 4, Female)

‘Members of the community will ask me embarrassing and derogatory questions if I reveal my status to them.’ (Participant 3, Female)

Generally speaking, most of the participants had disclosed only to family members, thus they had not really experienced discrimination from members of the community. One lost her boyfriend after disclosing but they all supported the notion of disclosing.

‘It is the people who can keep secret that should be told.’ (Participant 3, Female)

‘Disclosure should start with your family (‘charity begins at home’) before you disclose to outsiders.’ (Participant 2, Female).

‘It is not okay to disclose to everybody because they will run away.’ (Participant 1, Female)

The participants knew about ARVs; five of them were currently using them and they could mention some of their names; they also know that ARVs benefit the immune system and keep them healthy.

‘I was on stavudine, lamivudine and nevirapine but the stavudine was changed to zidovudine (because stauvudine was phased out). The benefits are that it makes me stronger and healthier. The second benefit is that it prevents me from breaking down, the third reason is that it increased my CD4 (from 732 to 914 recently).’ (Participant 6, Male)

The challenge experienced with taking the ARVs in the past were mainly due to side effects and timing. Two of the participants were on stavudine before it was phased out and experienced body fat redistribution; other side effects experienced by PLWHAs were rashes and black spots and changes in their menstrual cycles. Concerning side effects, some participants had their drugs changed by their doctors whilst others were reassured. They were able to cope with the side effects without missing doses because they understood the reason why adherence was important. At the time of the FGD none of the participants had issues concerning side effects.

‘The adherence challenge is taking the drug at the right time. The reason is because am usually in the bus at that period and without water. The presence of someone does not deter me from taking my drug, friends call me ‘drug addict’ because they see me taking drugs often but they do not know the drug I am taking. I just tell them that I am taking my fuel.’ (Participant 6, Male)

‘I have challenges when taking the drugs in the evening because I am usually out in the church during that period and there is no water.’ (Participant 2, Female)

On the need for adherence, the participants were of the opinion that adherence is important

‘To prevent the virus from ‘exploding’. (Participant 2, Female)

‘To prevent viral replication and increase CD4.’ (Participant 6, Male)

‘Adherence prevents resistance and the need for going from 1st line to 2nd or 3rd line regimen.’ (Participant 6, Male)

The coping mechanism for taking the drugs in the presence of people was to remove the drugs from their container and put them in an unlabelled pill box, which has allowed them the freedom of taking their drugs anywhere, anytime.

‘I set my phone alarm, and I remove the drug from its container and put it in a pill box from where I take the drug even in the presence of people.’ (Participant 2, Female)

‘I transfer the drug into a pill box and take them in the presence of people, taking the drug has prevented me from drinking beer.’ (Participant 6, Male)

## Discussion

In this study, most of the respondents were between the ages of 25 and 44 years (69.0%) and the mean age of respondents was 35.75 years ±10.74; there were more female respondents (64.5%) than male respondents; only 13.7% of the respondents had less than secondary school education, which suggests a high literacy level. Close to half of the respondents were married (42.6%); slightly over half of the respondents were Christians (56.9%) and the Yorubas (41.1%) were the predominant ethnic tribe.

The majority (92.9%) of the respondents had known their HIV status for more than six months and 83.2% had been attending the HIV clinic for minimum of six months. The majority of the respondents (71.6%) were on ARVs, and amongst this group 25.5% sometimes missed their medication. This is in line with a study carried out at a tertiary health facility in Edo State in 2008, which reported that 26.5% of PLWHAs sometimes miss their medication.^18, 19^

The main reason for respondents not taking their drugs was that they were tired of taking the medication (52.2%), followed by the presence of someone else (17.3%), travelling without taking along ARV medication (13.1%), feeling better and skipping doses (13.1%) and because of side effects (4.3%). This finding was corroborated by a study on AIDS patients from selected developed and developing countries (USA, Canada, Belgium, Brazil and Botswana) in 2006, which showed that being tired of taking ARV medication was the major reason why PLWHAs skip their doses on the average for all the countries (52.9%).^20^ However, a study in Rwanda in 2008 found that the main reason for respondents skipping their ARVs was because they travelled without taking along their medication (30.0%).^21^ This is in contrast with the result of the study done in Botswana in 2003, which established an association between side effects of ARVs and poor medication adherence.^22^ A similar study done in Kenya in 2009 showed that the main reason for missing doses was forgetting to take along their drugs when travelling (71.8%),^23^ and a study done in Baltimore, USA, in 2007, which involved urban youths (adolescents and young adults) of whom 56.9% were males, which revealed that half of the participants (50%) had missed medication doses for fear of disclosing their status.^11^

Most of the respondents who missed their medication for whatever reason because they travelled without their medication did so once in two months this is in contrast to the result of a study carried out in southwest Ethiopia in 2010 where only 19.8% of respondents travelled without their medication at least once in two months.^24^

Most of the respondents on ARVs (89.4%) were adherent to their medication, based on 7 days recall. The motivation for this high level of adherence, according to the respondents during the FGD, include availability of ARVs with minimal side effects, educating PLWHAs on the benefits and usefulness of ARVs in controlling the virus, and the fact that they remove the ARVs from the labelled containers and put them in unlabelled pill boxes, which gives them the freedom to take their drugs in the presence of other people. This level of adherence is higher than reported in the result of a study amongst PLWHAs in Benin city in 2008, which reported a medication adherence of 58.1%.^18^ One reason for the difference could be that 86.3% of respondents in this study had at least secondary school education, compared to 10.6% in the Benin study. However, a similar study carried out in Southwest Ethiopia in 2010 found that 95% of respondents on ARVs were adherent to their medication;^24^ the same was observed in a 2005 study carried out in Mombasa, Kenya.^25^ A 2006 study done in Chicago on medication adherence amongst PLWHAs who are of African-American origin reported that 70.6% of the respondents were adherent,^26^ and another medication adherence study done in Boston, USA, in 2006 gave a medication adherence of 90.0%.^27^

Amongst the various domains of stigma, public attitude stigma was very high (98%), whilst that of negative self image was the lowest, at 6.6%. This portrays stigma as being experienced externally and not internally, which is unlike what was observed in a study done in Los Angeles where public attitude stigma was experienced amongst 31% of the respondents, whilst negative self image stigma was experienced amongst 89% of respondents.^28^ This is also different from the result of a study carried out in 2005 in South India, where public attitude stigma was experienced amongst 72.2% of respondents whilst negative attitude stigma was experienced amongst 85.9% of respondents.^29^ However a study carried out in Kenya in 2008 found that 85% of the HIV+ respondents had experienced public attitude stigma,^30^ which is similar to the result obtained in this study. Furthermore, 37.1% of the respondents in this study had experienced personalised stigma, compared to 77.8% of respondents in a study carried out in Ogun State in 2008.^31^ The reason for the difference could be that the respondents in the Ogun state study were younger (18–35 years). Amongst the respondents in this study, 43.1% experienced disclosure stigma; this result corroborates that of a study done in sub-Saharan Africa in 2001 which revealed that 46.4% of respondents experienced disclosure stigma.^32^

Only 35% of the respondents were stigmatised; this low level is probably because, according to the FGD, the respondents practice limited disclosure (amongst close family members or loved ones or trusted friends). This result is consistent with a study done in the USA in 2009 which reports that one third (33.0%) of its study unit experienced stigma,^29^ but it is quite different from a study carried out in Ethiopia in 2008 where stigma prevalence was 86.4%.^13^

Increase in level of education was associated with a reduction in personalised stigma. Respondents who had tertiary education experienced less personalised stigma compared to those who had only primary education of (*χ*^2^ = 10.020, *p* = 0.018). This is in conformity with a 2003 study done in Tanzania which found that respondents with a higher level of education were less likely to experience personalised stigma.^33^ This could be due to the better living and working conditions and social support available to people with higher educational status.^13^ Amongst the ethnic groups, the Hausas experienced personalised stigma more than the other ethnic groups (*χ*^2^ = 9.969, *p* = 0.043).

Respondents who had been attending the HIV clinic for over 2 years experienced more personalised stigma than the others (*χ*^2^ = 16.052, *p* = 0.000). This suggests that stigma experienced by people living with HIV increases with time. In terms of ARV use, respondents who were on ARVs experienced more personalised stigma compared to PLWHAs who were not on ARVs (*χ*^2^ = 8.192, *p* = 0.004); this finding is in conformity with the result of a study carried out in Lesotho in 2009 which revealed an association between ARV use and personalised stigma.^34^ It is also similar to the result of a study carried out in Ethiopia in 2008 where it was found that PLWHAs on ART experienced personalised stigma more than those who were treatment naïve.^13^ Side effects of medication could be responsible for this; a study carried out in 2002 in three African countries, namely Ghana, Kenya and Rwanda, found that long term use of some ARVs can result in disfigurement, which can further add to stigma.^35^ Stavudine-based combinations cause lipodystrophy, whilst Nevirapine can cause rashes. Another reason why respondents on ARVs experience personalised stigma more than those not on medication could be because taking ARVs could precipitate a discriminatory attitude from the public. In a study conducted in China in 2007, 43.8% of respondents claimed that they had been treated differently by their neighbours and friends after their neighbours and friends knew that they were taking Highly Active Antiretroviral Therapy (HAART).^36^

A statistically significant association was observed between educational status, marital status, respondents’ awareness of their HIV status and disclosure stigma. This was also observed in a 2006 Niger Delta study which found that married respondents, respondents with no formal education and respondents who have known their status for less than 2 years were more likely to experience disclosure stigma.^37^ In this study, respondents with tertiary education were the least stigmatised in terms of disclosure stigma whilst respondents with primary education were the most stigmatised (*χ*^2^ = 9.211, *p* = 0.019). Married respondents experienced more disclosure stigma than the others (*χ*^2^ = 8.747, *p* = 0.033). This is similar to the findings of a study carried out South Africa in 2006.^38^ Respondents who had known their HIV status for between 6 months to 2 years experienced the most disclosure stigma (*χ*^2^ = 7.986, *p* = 0.018). This observation is consistent with a 2003 study carried out in Washington, USA, which discovered that the length of time after a PLWHA discovers his status is highly correlated with disclosure stigma.^39^ A statistically significant association was not observed between socio-demographic characteristics and negative self image stigma and public attitude stigma. This is similar to what was observed in a study carried out in Kenyatta, Kenya in 2007.^40^ The results of this study show that there was no statistically significant association between stigma and medication adherence, even though respondents who were not stigmatised were more adherent (90.8%) compared to respondents who experienced stigma (87.0%). This is consistent with a 2006 study done amongst African-Americans in Maryland, USA, where it was found that respondents who reported having missed medication doses also experienced higher stigma.^41^

## Practical implications

The results of this study revealed the complexity of HIV stigma. This is because the public attitude and the disclosure domain of stigma were high, whereas the personalised and the negative self image domain of stigma was low. This implied that the stigma experienced by people living with HIV is experienced externally and not internally. Also, the results of this study revealed that medication adherence by the PLWHAs is not a major problem as most of them were compliant.

## Limitations of the study

Patients who collect their medication by proxy from the clinic could not be assessed, thus they were excluded from the study.

## Recommendations

Based on findings from this study, the following recommendations are suggested:

1. The government should, in collaboration with NGOs and the mass media, educate the general public on HIV and AIDS with the view to reducing discrimination of PLWHAs and consequently reduce the public attitude stigma experienced by PLWHAs.
2. The government and NGOs should make stigma assessment an integral part of the monitoring and evaluation of HIV-related programmes geared towards PLWHAs. Programmes which reduce the stigma experienced by the participants should be adjudged to be more effective and efficient than other programmes with little or no effect on stigma reduction.
3. The PLWHAs should be encouraged to participate actively in support groups. This will reduce the public attitude stigma experienced by them in the long run.
4. More research should be done by researchers locally on the various stigma coping mechanisms available; PLWHAs should be encouraged and trained to practise such coping strategies to enable them to overcome the stigma associated with their condition.

## Conclusion

The result of this cross-sectional study carried out amongst adult PLWHAs attending the HIV clinic at General Hospital, Lagos, revealed that 35% of respondents experienced overall stigma. For the various domains of stigma assessed, almost all the respondents (98.0%) experienced public attitude stigma, 43.1% experienced disclosure stigma, 37.1% experienced personalised stigma, whilst a few of them experienced negative self image stigma (6.6%). This reveals that most of the stigma experienced by the respondents is external and not internal. The difference in proportion for the various domains of stigma confirms the complexity of HIV-related stigma.

The medication adherence of the respondents on ARV medication was very good (89.4%). Tiredness of taking medication was the main reason why respondents on antiretroviral medication skipped their doses (52.2%). Removing ARVs from labelled containers and putting them in unlabelled pill boxes was the adopted coping strategy.

Respondents having no formal education, the Hausa respondents, respondents on medication and respondents who have been attending the HIV clinic for over 2 years experienced personalised stigma the most. An association was also established between disclosure stigma and the education level of respondents, their marital status, as well as respondents’ awareness of their HIV status.
